# Implementation-effectiveness trial of systematic family health history based risk assessment and impact on clinical disease prevention and surveillance activities

**DOI:** 10.1186/s12913-022-08879-2

**Published:** 2022-12-06

**Authors:** R. Ryanne Wu, Rachel A. Myers, Joan Neuner, Catherine McCarty, Irina V. Haller, Melissa Harry, Kimberly G. Fulda, David Dimmock, Tejinder Rakhra-Burris, Adam Buchanan, Geoffrey S. Ginsburg, Lori A. Orlando

**Affiliations:** 1 Center for Applied Genomics and Precision Medicine, Department of Medicine, Duke University School of Medicine, Durham, NC USA; 2 Duke-NUS Medical School, Programme in Health Services and Systems Research, Singapore, Singapore; 3 Department of Medicine, Medical College of Wisconsin, Milwaukee, WI USA; 4 Center for Patient Care and Outcomes Research, Medical College of Wisconsin, Milwaukee, WI USA; 5 University of Minnesota Medical School, Duluth Campus, Duluth, MN USA; 6 Essentia Institute of Rural Health, Duluth, MN USA; 7 The North Texas Primary Care Practice-Based Research Network and Family Medicine, University of North Texas Health Science Center, Fort Worth, TX USA; 8 Rady Children’s Institute for Genomic Medicine, San Diego, CA USA; 9 Genomic Medicine Institute, Geisinger, Geisinger, PA USA; 10 All of Us Research Program, National Institutes of Health, Bethesda, MD USA

**Keywords:** Hybrid implementation-effectiveness, Risk assessment, Clinical decision support, Family health history, Precision medicine, Health belief model

## Abstract

**Background:**

Systematically assessing disease risk can improve population health by identifying those eligible for enhanced prevention/screening strategies. This study aims to determine the clinical impact of a systematic risk assessment in diverse primary care populations.

**Methods:**

Hybrid implementation-effectiveness trial of a family health history-based health risk assessment (HRA) tied to risk-based guideline recommendations enrolling from 2014–2017 with 12 months of post-intervention survey data and 24 months of electronic medical record (EMR) data capture. Setting:19 primary care clinics at four geographically and culturally diverse U.S. healthcare systems. Participants: any English or Spanish-speaking adult with an upcoming appointment at an enrolling clinic. Methods: A personal and family health history based HRA with integrated guideline-based clinical decision support (CDS) was completed by each participant prior to their appointment. Risk reports were provided to patients and providers to discuss at their clinical encounter. Outcomes: provider and patient discussion and provider uptake (i.e. ordering) and patient uptake (i.e. recommendation completion) of CDS recommendations. Measures: patient and provider surveys and EMR data.

**Results:**

One thousand eight hundred twenty nine participants (mean age 56.2 [SD13.9], 69.6% female) completed the HRA and had EMR data available for analysis. 762 (41.6%) received a recommendation (29.7% for genetic counseling (GC); 15.2% for enhanced breast/colon cancer screening). Those with recommendations frequently discussed disease risk with their provider (8.7%-38.2% varied by recommendation, *p*-values ≤ 0.004). In the GC subgroup, provider discussions increased referrals to counseling (44.4% with vs. 5.9% without, *P* < 0.001). Recommendation uptake was highest for colon cancer screening (provider = 67.9%; patient = 86.8%) and lowest for breast cancer chemoprevention (0%).

**Conclusions:**

Systematic health risk assessment revealed that almost half the population were at increased disease risk based on guidelines. Risk identification resulted in shared discussions between participants and providers but variable clinical action uptake depending upon the recommendation. Understanding the barriers and facilitators to uptake by both patients and providers will be essential for optimizing HRA tools and achieving their promise of improving population health.

**Trial registration:**

Clinicaltrials.gov number NCT01956773, registered 10/8/2013.

**Supplementary Information:**

The online version contains supplementary material available at 10.1186/s12913-022-08879-2.

## Background

Patient engagement is essential to achieving healthcare’s mission to improve health. However, engagement is widely variable, depending upon many factors such as socioeconomic status, risk perception, and trust. Risk perception reflects the patient’s beliefs about their disease risk and the potential of an intervention to reduce it. This relationship is described in the Health Belief Model (HBM), a highly validated model often used to guide the design of interventions [1]. The HBM posits that an individual’s course of action, as it relates to health, is informed by 1) perceived susceptibility, 2) perceived disease severity, 3) perceived intervention benefits, 4) barriers, 5) cues to action, and 6) self-efficacy. Interventions designed to affect one or more of these components can enhance the likelihood that an individual will take a specific health-related action [2, 3].

One intervention, increasingly being used to improve individual and population health, is the health risk assessment (HRA). HRAs allow individuals to be stratified into categories based on their risk for developing a specific condition. However, quantifying disease risk alone is not enough to improve health. The last decade has seen an explosion of risk-based guidelines – guidelines that recommend different disease screening/prevention strategies for different risk levels. Tying the level of risk to a specific risk mitigation strategy closes the loop and leads to actions that have the potential to improve health. But to achieve the full benefit, HRAs need to: 1) be implemented systematically (i.e., everyone should be encouraged to complete it); and 2) enhance uptake of the risk-based guideline recommendations. Currently, systematic HRAs are not widely utilized, largely due to the complexity of the data needed to run the HRA, lack of structures to support systematic assessment, and failure to adapt to a specific setting’s needs [4]. Several HRAs have been developed and trialed in single institutions with evidence of significant increase in risk identification but none have been implemented more broadly to understand barriers and facilitators in diverse healthcare systems and populations thus there is limited understanding of clinical impact [5–7].

In this paper, we report the clinical impact of a systematic family health history (FHH)-based HRA on 1) provider referral for and 2) patient performance of risk-based guideline recommendations across four diverse healthcare systems. The HBM was used to guide the development, deployment, and evaluation of the HRA intervention. We have previously published this study’s implementation outcomes [8, 9] and the potential impact of systematic HRA on population health [10]. This study was funded by the National Institutes of Health as part of the Implementing Genomics in Practice network [11].

## Materials and methods

We performed a pragmatic real-world Type III hybrid implementation-effectiveness trial to evaluate the impact of a FHH-based HRA on clinical uptake of risk-based guideline recommendations by providers and patients [12, 13].

### Setting

We recruited participants from nineteen primary care clinics in four U.S. healthcare systems (Duke Health, Essentia Health, Medical College of Wisconsin, University of North Texas Health Science Center) that varied in their setting (rural/urban), patient population (predominantly White/Hispanic/Black), and infrastructure (public/private; academic/non-academic). Recruitment was from 2014 – 2017.

### Participants

All English and Spanish speaking patients with upcoming appointments at participating primary care clinics were eligible to participate.

### Intervention

The patient-facing web-based systematic HRA platform, MeTree, was designed using the HBM [14]. It integrates personal characteristics (e.g., blood pressure, medical history) and FHH to generate personalized CDS reports for disease prevention and surveillance based on risk-based U.S. guidelines. CDS reports are tailored to each user (patient and provider) to enhance shared decision making around risk and recommended actions to mitigate that risk. A complete description of MeTree has been published previously [15], although its functionality has been enhanced over time [12]. It currently collects information on 98 medical conditions and provides CDS regarding screening and prevention strategies for 30, in both English and Spanish.

### Study procedures

Providers at participating clinics indicated their interest (or not) in the study. If interested, their consentable English and Spanish speaking adult patients were offered enrollment when scheduling an appointment. Following consent, participants completed a web-based survey and the HRA. Patient-oriented CDS reports were generated in real-time for participants to view and download. Provider-oriented CDS reports were uploaded to the patient’s electronic medical record (EMR) as a clinical note, and providers notified via EMR messaging. Participants completed additional web-based surveys at 3 and 12 months post-intervention. At the end of the study, EMR data was extracted to assess actions taken relevant to the CDS’s risk-based guideline recommendations. The study protocol [12] and study flow [8] are published.

### Measures

Participant data captured in the HRA included: age, race/ethnicity, and medical conditions (with age of diagnosis). To adequately assess risk, participants were required to enter FHH for parents, and maternal and paternal grandparents, but were encouraged to add as much information about additional relatives as they wished. Data captured by the HRA for relatives included: current age (or age and cause of death if applicable), and medical conditions (with age of diagnosis).

Data generated by the HRA included risk calculations and each participant’s guideline-based risk management recommendations. In this paper, we limited the analysis to those recommendations with a clear link between screening/prevention recommendation and action (Table 1). For example, we excluded recommendations for hereditary hemochromatosis screening with a ferritin due to how frequently it’s ordered for other clinical reasons, e.g. anemia. Risk management recommendations are categorized into two risk levels: 1) monogenic, reflecting risk for highly penetrant high risk gene variants (e.g., pathogenic *BRCA1* variant in Hereditary Breast and Ovarian Cancer Syndrome), and 2) familial, indicating risk significantly higher than the general population but not as high as monogenic.

**Table 1:** MeTree conditions and associated clinical decision support

**Condition**	**Guideline-based risk management recommendation**
**Monogenic RISK**	
**• Hereditary cancer syndromes (*N* = 20)**	• **Genetic counseling** for comprehensive cancer risk assessment & management
**• Hereditary cardiovascular syndromes (*N* = 9)**	• **Genetic counseling** for comprehensive inherited cardiac disease risk assessment & management
**• Familial hypercholesterolemia**	• **Genetic testing** to screen for Familial Hypercholesterolemia
**• Thrombosis**	• **Genetic testing for inherited thrombophilia** • **Genetic counseling** for comprehensive inherited thrombophilia risk assessment & management
**Familial risk**	
**• Breast cancer**	• **Breast cancer surveillance via annual breast MRI and mammography** • **Discuss chemoprevention** for breast cancer (tamoxifen or raloxifene) • **Discuss chemoprevention** for breast cancer (tamoxifen)
**• Colon cancer**	• **Colonoscopy**, with frequency based on number, size and histology of polyps • **Colonoscopy every 1–2 yrs** with biopsies for dysplasia, beginning 8 yrs after onset of pancolitis, or 12–15 yrs after onset of left-sided colitis • **Early colorectal cancer surveillance** (beginning at age 40) • **Early colorectal cancer surveillance** (beginning at age 45) • **Early and more frequent colonoscopies** (every 5 years beginning at age 40 or 10 yrs younger than the earliest diagnosis in the family, whichever comes first)

Patient surveys administered at baseline, 3, and 12 months (Appendices 1, 2, and 3) measured health-related activation with the validated Patient Activation Measure (PAM) [16] and questions about: 1) topics discussed with providers; 2) satisfaction with those discussions on a Likert scale ranging from 1-very poor to 5-superior; and 3) intentions to pursue provider recommended cancer screening on a Likert scale ranging from 1- “I do not intend to pursue cancer screening” to 5- “I have been obtaining cancer screening exactly as my doctor tells me for over a year.”

A provider survey (Appendix 4) administered at study end measured intervention impact, acceptance, and barriers to recommendation uptake using yes/no and Likert scales. Items included “I understand risk scores better now than when the study started” (1-strongly disagree to 5-stronlgy agree), “Would it be helpful to have a relationship with a genetic specialist?” (yes/no/not sure), and “Did generating risk scores negatively impact the clinic workflow or patient flow in your clinic?” (yes/no). Providers also identified specific recommendations they could not complete (e.g. breast MRI, genetic counselling) due to resource and process barriers and how having risk scores changed their practice.

We analyzed EMR data to evaluate actions taken prior to and 24 months following the intervention; specifically: breast MRI, colonoscopy, fecal immunohistochemistry tests (FIT), fecal occult blood tests (FOBT), sigmoidoscopy, tamoxifen/raloxifene, and genetic counseling (GC).

### Outcomes

The primary outcome was provider and patient uptake of risk-based guideline recommendations. Provider uptake was defined as placing an order for a recommended action. Participant uptake was defined as completing the recommended action, when a provider ordered it. Factors affecting participant uptake were evaluated using the HBM and provider uptake using the Consolidated Framework for Implementation Research (CFIR) (http://cfirguide.org/) [13, 17]. CFIR is a comprehensive and unified ontology of overarching implementation themes drawn from published models. It comprises 39 constructs organized across five domains: 1) Intervention Characteristics; 2) Outer Setting; 3) Inner Setting; 4) Characteristics of Individuals; and 5) Process, which interact in dynamic and complex ways. Provider uptake is reflected in the Innovation Characteristics domain by the evidence strength, relative advantage, and design quality constructs; the Outer Setting domain by the external policies construct; the Inner Setting domain by the compatibility, relative priority, and access to knowledge constructs; and the Characteristics of Individuals domain by the intervention knowledge/beliefs, self-efficacy, and individual stage of change constructs.

As described earlier, we restricted our evaluation to guideline-based recommendations with actions attributable to the CDS (Table 1) and compared pre-intervention actions to post-intervention. Early colonoscopy uptake assessments were limited to participants < 50 years old, as those ≥ 50 were eligible for routine screening (per guidelines at the time of the study). However, in those with early and more frequent (EMF) CRC screening recommendations, we assessed the more frequent component across all ages. To account for delays in scheduling/access and differences in appropriate screening intervals, timeframes for pre-intervention assessments varied by recommendation. For breast MRI the timeframe was two years prior since it is performed annually [18]. For CRC screening, assessment timeframes differed between early and EMF recommendations. EMF recommendations specify a colonoscopy at least every five years [19]; therefore, the assessment timeframe was six years prior. Early recommendations specify screening with any modality starting before age 50. However, the recommended start age varies depending upon the age of onset in the affected relative, and the frequency varies depending on the modality (e.g. FIT or FOBT annually or colonoscopy every 10 years); therefore the pre-intervention assessment timeframe was tailored to the prior screening modality and the guideline recommended start age.

### Statistical analysis

Descriptive statistics summarized participant demographics, participant-provider discussions, participant and provider uptake of guideline recommendations, and participant cancer screening survey responses. Fishers exact test assessed independence between participant demographics and CDS guideline risk level (monogenic, familial, or any risk), specific risk recommendation, provider uptake of recommendations, and participant uptake of recommendations. Similarly, Fisher’s exact test assessed independence between risk recommendation and participant-provider discussions, as well as the participant’s plan for cancer screening. Differences in the proportion of provider or participant uptake of cancer and non-cancer related GC recommendations were evaluated using a two-sample test of proportions.

## Results

**Fig. 1:**
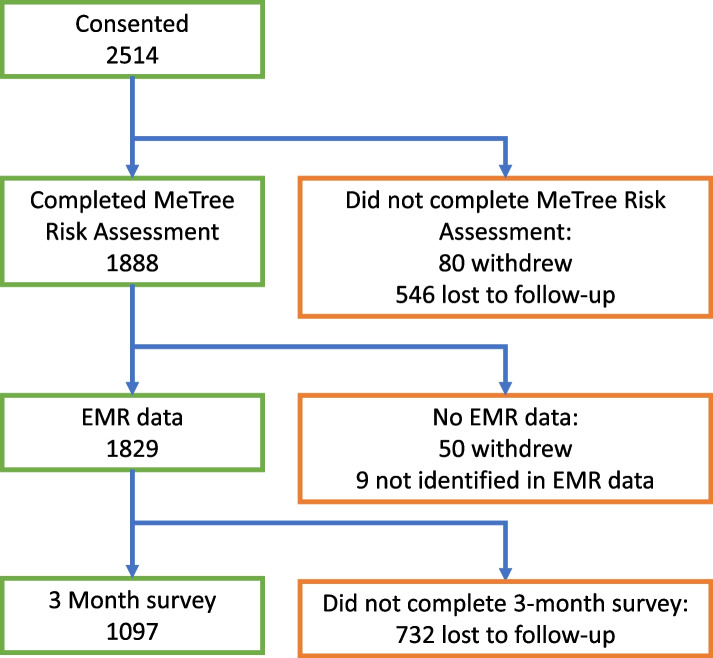
Enrollment and study flow

We enrolled 2,514 participants; 1,889 (75.1%) completed the HRA. Of these 1,829 (96.8%) had EMR data and were included in the analysis; 1,097 (60.0%) completed the 3-month survey (Fig. 1). Demographics of the overall population, those in each risk level (familial, *N* = 278; monogenic, *N* = 543), and any risk level (any CDS guideline recommendation, *N* = 762) are reported in Table 2. The majority (71.2%) of recommendations were for genetic counseling (monogenic risk). All risk levels were statistically similar to the underlying study population racially and ethnically, but were statistically different in gender and insurance type (familial *P* < 0.001 for both, monogenic *P* < 0.001 for gender and 0.003 for insurance, any recommendation *P* < 0.001 for both). This bias towards females was expected given that recommendations for breast and ovarian cancer primarily (but not exclusively) affect females. Insurance type though statistically significant was only clinically significant in the familial risk group. The any recommendation group and the familial risk group were also younger (*P* < 0.001 for both) and the familial risk group was more educated (*P* = 0.003).

**Table 2:** Participant Demographics

Demographic	Total population	Familial Risk recommendationa N (%)	Monogenic recommendationa N (%)	Any increased risk recommendationa N (%)
N	1829	278 (15.2)	543 (29.7)	762 (41.7)
Age (SD)	56.2 (13.9)	51.6 (11.4)b	55.3 (13.6)	54.6 (13.1) b
Female	1273 (69.6)	233 (83.8) b	424 (78.1) b	558 (79.4) b
Race				
• Asian	20 (1.1)	3 (1.1)	5 (0.9)	6 (0.9)
• Black	139 (7.9)	20 (7.5)	46 (8.7)	56 (8.2)
• White	1561 (88.7)	238 (89.5)	465 (87.7)	602 (88.5)
• Other	39 (2.2)	5 (1.9)	14 (2.6)	16 (2.4)
Hispanic Ethnicity	33 (2.3)	3 (1.4)	12 (2.8)	13 (2.4)
Insurance				
• Private	1250 (69.1)	227 (83.2) b	380 (70.4)	511 (73.3) b
• Medicare	513 (28.4)	38 (13.9) b	138 (25.6)	161 (23.1) b
• Medicaid	31 (1.7)	3 (1.1)	12 (2.2)	13 (1.9)
• Other	14 (0.8)	5 (1.8) b	10 (1.9) b	12 (1.7) b
College education or more	1394 (76.2)	192 (69.1) b	408 (75.1)	518 (73.7) b

^a^Risk recommendations for outcomes reported in this manuscript (i.e. familial recommendation = breast MRI, chemoprevention, earlier CRC screening, EMF CRC screening; monogenic recommendation = genetic counselling.)

^b^*p*-value < 0.05 for comparison of given category to total population

### Post-intervention participant-provider discussions

Participants with any guideline recommendation for risk management were more likely to discuss their disease-specific risk and risk prevention/mitigation strategies with their provider than those without (Table 3); however, all participants were satisfied with their provider conversations regardless of receiving a guideline recommendation (958 [90.0%] average to superior satisfaction and 107 [10.0%] below average to very poor). Notably, participants with a monogenic guideline recommendation (genetic counseling) who discussed their risk with their provider were more likely to be referred to genetic counseling (*N* = 12/27 (44.4%) with a provider discussion; *N* = 17/284 (5.9%) without a discussion, *P* < 0.001), while the same was not true for those with a familial guideline recommendation (breast MRI orders: *N* = 4/6 [66.6%] with provider discussion versus *N* = 9/28 [32.1%] without discussion, *P* = 0.17; CRC screening orders: *N* = 14/46 [30.4%] with provider discussion versus *N* = 7/34 [20.6%] without discussion, *P* = 0.44). Interestingly, 147 participants reported discussing breast cancer chemoprevention with their provider though only 18 received a chemoprevention recommendation. However, since no providers ordered it, the discussions had no impact on uptake.

**Table 3:** Participant-Provider discussions

Recommendationa	Participants reporting discussion of relevant topic N (%, 95% CI)b	Participants with relevant CDS recommendation N (%, 95% CI)c	Participants without relevant CDS recommendation N (%)d	*P*-value
Breast MRI	161 (14.7, 12.7–16.9)	13 (38.2, 22.7 – 56.4)	148 (13.9, 11.9 – 16.2)	< 0.001
Chemoprevention	147 (13.4, 11.5—15.6)	18 (30.5, 19.5 – 40.0)	129 (12.4, 10.5 – 14.6)	< 0.001
Colonoscopy	156 (14.2, 12.2- 16.5)	21 (26.3, 17.3 -37.5)	135 (13.3, 11.3 – 15.6)	0.004
Genetic counselling overall	44 (4.0, 3.0—5.4)	27 (8.7, 5.9 – 12.5)	17 (2.2, 1.3 – 3.5)	< 0.001
GC for breast cancer risk	176 (16.0, 13.9, 18.4)	36 (24.8, 18.2 – 32.8)	140 (14.7, 12.5 – 17.2)	0.003
GC for colon cancer risk	192 (17.5, 15.3—19.9)	32 (28.6, 20.6 – 38.0)	160 (16.2, 14.0 – 18.7)	0.002

^a^Discussion topics relevant for each recommendation as follows: breast MRI (discussion of breast MRI, overall breast cancer [BC] risk), chemoprevention (discussion of chemoprevention, overall BC risk), colonoscopy (discussion of overall colon cancer risk), GC (discussion of GC), GC for BC risk (discussion of GC, overall BC risk), GC for colon cancer risk (discussion of GC, overall colon cancer risk)

^b^% of total participants who responded to the survey (1097)

^c^% of all participants who responded to the survey and had the specified CDS recommendation

^d^% of all participants who responded to the survey and did not have the specified CDS recommendation

### Uptake of familial risk recommendations

The mean age of those receiving a familial guideline recommendation was 51.6 (SD 11.4); 83.8% were female with a racial distribution reflective of the underlying population (Table 2). Table 4 describes provider and participant uptake of risk-based guidelines. There was an increase in provider uptake of breast MRI and CRC related recommendations, but as in our prior study, no provider ordered breast cancer chemoprevention medications (either before or after the intervention) [20]. The strongest effect for both patients and providers was seen in colon cancer screening. For providers there was a 7.4% increase in guideline uptake in the subset of participants age < 50 years old, and 7.6% in those aged ≥ 50 years old with an EMF colonoscopy recommendation. For patients there was a 10.8% increase in guideline uptake in the subset of participants age < 50 years old, and 18.7% in those aged ≥ 50 years old with an EMF colonoscopy recommendation.

**Table 4:** Uptake of risk-based guideline recommendations

Guideline Recommendation	Familial risk recommendation (% of total population, 95% CI)	Pre-intervention Uptake of risk-based guidelines		Post-intervention Uptake of risk-based guidelines	
		Provider uptake (%, 95% CI)a	Patient uptake (%, 95% CI)b	Provider uptake (%, 95% CI)a	Patient uptake (%, 95% CI)b
Any familial recommendation	278 (15.2, 13.6—16.9)	69 (22.6)	48 (69.6)	83 (27.2)	69 (81.2)
• Breast MRI	54 (3.0, 2.2—3.9)	3 (5.5, 1.4 -16.3)	3 (100, 31.0—100)	6 (11.1, 4.6 – 23.3)	4 (66.6, 24.1 – 94.0)
• Chemoprevention	106 (5.8, 4.8—7.0)	0 (0, 0 – 4.4)	0	0 (0, 0 – 4.4)	0
• Early or EMF CRC Screening (for patients < 50)	67 (3.7, 2.9—4.7)	19 (28.4, 18.3 – 40.9)	13 (68.4, 43.5 – 86.4)	24 (35.8, 24.7 – 48.5)	19 (79.2, 57.3 – 92.0)
• EMF (for patients > = 50)	78 (4.3, 3.4—5.3)	47 (60.3, 48.5 – 71.0)	32 (68.1, 52.7 – 80.5)	53 (67.9, 56.3 – 77.8)	46 (86.8, 74.0 – 94.1)
Monogenic recommendation	543 (29.7, 27.6—31.9)	n/ac	n/ac	51 (9.4, 7.1 – 12.2)	34 (66.7, 52.0 – 78.9)

^a^% of total population that received the specified recommendation

^b^% of participants who complete the specified recommendation of those with provider uptake

^c^GC referrals were abstracted manually from the EMR and were not captured pre-intervention

### Uptake of monogenic risk recommendations

Five hundred and forty three participants received a monogenic guideline recommendation (i.e., genetic counseling/genetic testing) (Table 2). Providers ordered GC referrals for 51 (9.4%) of those who met guideline criteria (Table 4). There was no difference in provider uptake of GC recommendations by institution (*P* = 0.15). When controlling for institution, younger participants and women were more likely to be referred (mean age of those referred = 50.3 [SD 11.8] vs. not referred = 55.9 [SD 13.7], *P* = 0.005, and 10.6% of women vs. 5.0% of men were referred, *P* = 0.05). There was otherwise no difference in demographics between those referred for GC and those not. Of the 51 participants referred to GC, 34 (66.6%) attended. There were no statistically significant differences in demographics or participant institutions for those who attended GC versus those who did not.

The majority of monogenic recommendations were for cancer-related syndromes (*N* = 364, 74%); 58 participants had recommendations for both cancer and non-cancer syndromes. Among those with only one GC recommendation, providers were more likely to refer participants with a cancer related recommendation than a non-cancer one (cancer = 36, 14.5% vs. non-cancer = 8, 6.6%, *P* = 0.01). There were no statistically significant differences in participant attendance of GC, though the low number of non-cancer referrals limits statistical inference (cancer = 26 (89.7%), vs. non-cancer = 3 (10.3%) *P* = 0.14).

### Patient activation

Patient activation based on the PAM score was high at baseline (mean PAM = 70.9 [SD 14.0]), and there was no difference between participants who received an increased risk recommendation and those who did not. A score of 70.9 ± the SD indicates Stage 3 or 4 on the PAM (i.e. the patient is “beginning to engage in recommended health behaviors” or is “proactive and engaged in recommended health behavior”) [21]. Given this homogenously high PAM score, it is not surprising that it was not associated with provider’s orders for or participant’s completion of guideline-based risk management recommendations.

For those completing the three-month follow-up survey (*N* = 1,097, 60%), participants with breast MRI or GC recommendations were more likely to report that they had already completed their cancer screening or intended to undergo cancer screening within the next month (*P* = 0.003 for breast MRI recommendation vs no breast MRI recommendation; and *P* < 0.001 for GC recommendation vs no GC recommendation). The same was not observed for those with CRC screening recommendations vs those without (*P* = 0.69).

### Provider experience and barriers to uptake

Following the intervention, providers understood risk scores better (20/42, 47.6%), rarely disagreed with a recommendation (2/40, 5.0%), felt patient communication was enhanced (28/40, 70%), reported it did not negatively impact their workflow (33/41, 80.5%), and would recommend standardized patient-facing risk assessment to their peers (38/42, 90.5%). They felt implementation would benefit from having a relationship with a genetics specialist (31/41, 75.6%) and reported process and resource related barriers to completing some recommendations (breast MRI *N* = 6, genetic counseling/testing *N* = 3, breast cancer chemoprevention *N* = 0, EMF CRC screening *N* = 0).

## Discussion

In this multi-institution implementation-effectiveness trial of a FHH-based risk assessment intervention, we consistently found a significant portion (41.2%) of the primary care population meets guideline criteria for enhanced surveillance due to familial risk of breast or colon cancer, and genetic counseling for monogenic hereditary syndromes. Importantly this finding was consistent across all four healthcare systems despite their differences in geography and population characteristics. However, clinical uptake varied by recommendation (highest for early and more frequent colonoscopies and lowest for breast cancer chemoprevention). Interestingly uptake also varied within the genetic counseling subgroup, where cancer genetic counseling had greater uptake than non-cancer, though there was no difference in referral rates by race, insurance, or education level. Given that genetic counseling is a severely constrained resource, the relative paucity of non-cancer genetic counselors to cancer genetic counselors may be exacerbating this disparity.

The CFIR provides a framework for understanding factors that affect provider uptake [17]. Providers felt the intervention improved clinical care (i.e. improved understanding of risk scores and enhanced patient communication) and would recommend it to their peers, suggesting that implementation successfully addressed the CFIR Intervention Characteristics’ constructs evidence strength & quality and relative advantage; and Characteristics of Individuals’ constructs knowledge & beliefs about the intervention and self-efficacy for the intervention as a whole. However, some guideline recommendations had poorer uptake compared to others (e.g. chemoprevention vs colon cancer screening). These differences can largely be explained by inadequate networks and communication processes (Inner setting domain) such as the need for closer relationships with a genetics specialist, and external policies and incentives (Outer setting domain) such as access to breast MRIs and genetic counselors. These are reflected in provider responses to resource related barriers which suggested breast MRI and genetic services were challenging, while colonoscopy related resources were not. This makes sense when considering usual practices in primary care where routine colonoscopies are common and therefore processes and procedures are already in place, whereas breast MRI and genetic counseling are not. One caveat is the special case of breast cancer chemoprevention, which continues to be challenging. Studies have repeatedly shown that primary care providers are uncomfortable prescribing tamoxifen or raloxifene for prevention of breast cancer and there seems to be no change in their attitude towards its risks and benefits over the last 20 years [22–24]. Even with this study’s clinical decision support highlighting the guideline and patients initiating discussions, there were no orders placed. And while none of the providers indicated that chemoprevention had a resource related barrier, this could be either because prescribing medications is a common and straightforward practice and/or it is not a barrier if they do not intend to prescribe the medication. Although implementation barriers to chemoprevention were not explicitly evaluated in this study, previous studies point to concerns about medication adverse events [22, 24, 25]. Therefore, employing implementation strategies related to the CFIR constructs ‘knowledge & beliefs about the intervention’ and ‘individual stage of change’ (in the Characteristics of Individuals domain) may be an effective next step to enhance uptake of this guideline.

The HBM provides a framework for understanding factors that affect patient uptake. In this study we found that participants at increased risk for a disease were more likely to discuss their risk and options for disease prevention with their provider and indicated plans to pursue screening as recommended by their provider, which is consistent with our prior study [26]. These findings reflect a relationship between the FHH-based risk assessment intervention and components of the Health Belief Model (HBM)—understanding disease risk (perceived susceptibility) and learning of risk mitigation strategies (perceived intervention benefits) informed health related actions (uptake of guideline recommendations). In addition, participant-provider discussions increased provider uptake of genetic counseling recommendations (more referrals were made) but not familial risk recommendations, suggesting that discussions affect the ‘perceived disease severity’ component of the HBM. This is an interesting finding given that those with monogenic recommendations (genetic counseling) are in fact at much higher disease risk than those with familial recommendations. Ideally, however, provider discussions related to familial risk, which is well above general population risk, would also lead to an increase in uptake. Since there was an overall increase in uptake of familial guideline recommendations after receiving the intervention, the recommendation itself has an impact but the discussion does not enhance that uptake. In part the lack of statistical significance may be attributable to small overall numbers, but future studies could consider incorporating implementation strategies focused on enhancing discussions related to the HBM’s perceptions of disease severity component to further optimize uptake.

While we were pleased to see the increased uptake of guideline-based risk management strategies in this trial, there is still more to be done to optimize uptake. First, there are process barriers that can now be more fully addressed with recent advances in CDS-EMR integration. In our study, provider CDS was uploaded to the participant’s chart as a PDF document in the EMR’s notes tab. While providers were manually notified at the time of CDS upload, notifications and participant clinical visits were asynchronous. Thus, many providers may have missed reviewing the CDS at the time of the visit- when discussions would be most effective. In contrast, in our prior study when clinical practices were still using paper records, a printed copy of the CDS report was paper clipped to the top of the patient’s chart and handed to the provider at the time of the clinical visit. We saw greater uptake of recommendations with this workflow than our current study’s, which highlights the need for providing the right information at the right time (one of the key principles of optimal CDS) [27]. To fully achieve the potential for systematic risk assessment in an electronic world, family history should be collected as structured data and risk assessment tightly integrated into clinical and patient EMR workflows. Unfortunately, no EMR currently has this capability, and given the many challenges to collecting high quality FHH none are likely to in the near future. However, EMRs have made significant advances in alert functionality through best practice alerts that automate right time, right patient, right information notifications. In light of what the EMR can and cannot do to support systematic risk assessment, the most efficient path for the immediate future will likely be integrating third party applications with the EMR, a possible but still somewhat difficult task [28].

Second, we should not forget that implementing a risk management recommendation is not a decision made by the provider alone. Uptake requires shared decision making between the provider and the patient and thus orders are not usually placed without the buy-in of the patient. This is where tenets of the HBM could be more fully integrated. Identifying risk, while an essential first step, is not sufficient alone. HRAs can work to optimize just-in-time education on disease risk (“perceived susceptibility”) and potential severity (“perceived disease severity”), as well as risk mitigation and surveillance options (“perceived intervention benefits and barriers”) so that patients understand the risks and benefits of recommended actions. While our intervention addressed each of these components and the study’s findings suggest it had an impact, better integration and further refinement of these components will likely promote greater patient self-efficacy and further empower them to make health related decisions.

There are limitations to this study’s data analysis and interpretations. While we recruited from diverse healthcare systems to facilitate greater racial and socioeconomic diversity, the study population was largely White, privately insured, and highly educated. Therefore, our uptake outcomes are reflective of diverse settings but not of diverse participants. The generalizability of our findings to diverse populations will thus be limited. We are currently initiating a trial in clinics with primarily rural, underserved, and minority patients that we hope will address the remaining questions about uptake and effectiveness in unique populations. In addition, measuring uptake with EMR data has its own constraints and challenges. In particular, missingness is hard to quantify and the lack of context to the data requires a certain level of inference as to why a test was ordered (e.g., was a CRC screening ordered due to the CDS or because of a clinical symptom). To minimize incorrect attribution, we limited our analysis to orders that are most commonly associated with the guideline recommendation and within a set time window of the intervention [22].

## Conclusions

This study builds on our prior work showing that systematic risk assessment can be successfully implemented across diverse healthcare systems [8, 9], has the potential to significantly impact population health as evidenced by increased risk identification [10, 29], and can enhance guideline based risk mitigation efforts that are widely accepted to improve health outcomes. It is also evident that further HRA development is needed to continue to close the gap between risk identification and risk mitigation.

## Supplementary Information

**Additional file 1.**

**Additional file 2.**

**Additional file 3.**

**Additional file 4.**

## Data Availability

Primary data is available upon request from the study principal investigator, Dr. Orlando. Code is available upon request from the study principal investigator, Dr. Orlando.

## Abbreviations

HBM: Health Belief Model
HRA: Health risk assessment
FHH: Family health history
CDS: Clinical decision support
EMR: Electronic medical record
PAM: Patient activation measure
GC: Genetic counselling
FIT: Fecal immunochemical test
FOBT: Fecal occult blood test
CFIR: Consolidated Framework for Implementation Research
EMF: Early and more frequent
CRC: Colorectal cancer
