# Supplementary material for: Implementation-effectiveness trial of systematic family health history based risk assessment and impact on clinical disease prevention and surveillance activities
Source: BMC Health Serv Res. 2022 Dec 6;22:1486. doi: 10.1186/s12913-022-08879-2 (PMC9727967; doi:10.1186/s12913-022-08879-2)
Supplement: Supplementary file 1 — Additional file 1. [file 12913_2022_8879_MOESM1_ESM.doc]

**HEALTH LITERACY**

1. How confident are you filling out forms by yourself

□ always

□ often

□ sometimes

□ occasionally

□ never

**MEDICATION ADHERENCE**

In order for medication to work, people have to take it as prescribed. For one reason or another, many people can’t or don’t always take all of their medication as prescribed. We want to know how often you have missed your _________ pills. When responding, please think about your pills only.

| **Over the past 7 days…** | **None of the time** | **A little of the time** | **Some of the time** | **Most of the time** | **Every**  **time** |
| --- | --- | --- | --- | --- | --- |
| 2. I missed my medicine. | ○ | ○ | ○ | ○ | ○ |
| 3. I skipped a dose of my medicine. | ○ | ○ | ○ | ○ | ○ |
| 4. I did not take a dose of my medicine. | ○ | ○ | ○ | ○ | ○ |

**READINESS TO CHANGE**

Please CHECK **ONLY** **ONE** response for each question.

**5.** Which of the following describes your behavior for weight reduction?

1 I do not intend to lose weight.

2 I am seriously considering losing weight in the next 6 months

3 I have a specific plan to lose weight, beginning in the next month.

4 I am working to lose weight right now

5 I intentionally lost weight recently, and I am actively working to maintain my current weight

**6.** Which of the following describes your behavior for exercise?

1 I do not intend to increase my exercise.

2 I am seriously considering increasing my exercise to 30 minutes a day in the next 6 months

3 I have a specific plan to increase my exercise to 30 minutes a day, beginning in the next month.

4 I started exercising 30 minutes a day in the past year

5 I started exercising 30 minutes a day over a year ago.

**7.** Which of the following describes your behavior for quitting smoking?

1 I do not intend to quit smoking

2 I am seriously considering quitting smoking in the next 6 months

3 I have a specific plan quit smoking, beginning in the next month.

4 I quit smoking in the last year, and have stayed quit

5 I quit smoking more than a year ago, and have stayed quit

6 I never smoked

**8.** Which of the following describes your behavior for healthier eating?

1 I do not intend to eat healthier.

2 I am seriously considering eating healthier in the next 6 months

3 I have a specific plan to eat healthier, beginning in the next month.

4 I am eating healthier in the last year.

5 I intentionally started eating healthy over a year ago and am still eating healthier

**9.** Which of the following describes your behavior for stress management?

1 I do not intend to do anything to reduce stress.

2 I am seriously considering trying to reduce my stress level in the next six months.

3 I have a specific plan to reduce stress, starting in the next month.

4 I am working to reduce stress right now, and have been for less than a year.

5 I made changes in my life to reduce stress over a year ago and I am working to maintain my

current stress level.

**10.** Which of the following describes your behavior to obtain cancer screening exactly as your doctor tells you to?

1 I do not intend to obtain cancer screening exactly as my doctor tells me to.

2 I am seriously considering obtaining cancer screening exactly as my doctor tells me to, beginning

in the next 6 months.

3 I have a specific plan to obtain cancer screening exactly as my doctor tells me to, beginning in the next month.

4 I have been obtaining cancer screening exactly as my doctor tells me to for less than a year.

5 I have been obtaining cancer screening exactly as my doctor tells me to for over a year.

# **PATIENT ACTIVATION MEASURE**

# Below are some statements that people sometimes make when they talk about their health.

# Please indicate how much you agree or disagree with each statement

# as it applies to you personally by checking your answer.

# ***Your answers should be what is true for you and not just what you think the doctor wants you to say.***

If the statement does not apply to you, check “N/A.”

| (check one response for each item) | Disagree strongly | Disagree | Agree | Agree strongly | N/A |
| --- | --- | --- | --- | --- | --- |
| **11.** When all is said and done, I am the person who is responsible for managing my health | 1🞏 | 2🞏 | 3🞏 | 4🞏 | 5🞏 |
| **12.** Taking an active role in my own health care is the most important factor in determining my health and ability to function | 1🞏 | 2🞏 | 3🞏 | 4🞏 | 5🞏 |
| **13.** I am confident that I can take actions that will help prevent or minimize some symptoms or problems associated with my health | 1🞏 | 2🞏 | 3🞏 | 4🞏 | 5🞏 |
| **14.** I know what each of my prescribed medications do | 1🞏 | 2🞏 | 3🞏 | 4🞏 | 5🞏 |
| **15.** I am confident that I can tell when I need to go get medical care and when I can handle a health problem myself | 1🞏 | 2🞏 | 3🞏 | 4🞏 | 5🞏 |
| **16.** I am confident I can tell a doctor concerns I have even when he or she does not ask | 1🞏 | 2🞏 | 3🞏 | 4🞏 | 5🞏 |
| **17.** I am confident that I can follow through on medical treatments I may need to do at home | 1🞏 | 2🞏 | 3🞏 | 4🞏 | 5🞏 |
| **18.** I understand the nature and causes of my health problems | 1🞏 | 2🞏 | 3🞏 | 4🞏 | 5🞏 |
| **19.** I know the different medical treatment options available for my health conditions | 1🞏 | 2🞏 | 3🞏 | 4🞏 | 5🞏 |
| **20.** I have been able to maintain the lifestyle changes for my health that I have made | 1🞏 | 2🞏 | 3🞏 | 4🞏 | 5🞏 |
| **21.** I know how to prevent problems with my health | 1🞏 | 2🞏 | 3🞏 | 4🞏 | 5🞏 |
| **22.** I am confident I can figure out solutions when new situations or problems arise with my health | 1🞏 | 2🞏 | 3🞏 | 4🞏 | 5🞏 |
| **23.** I am confident that I can maintain lifestyle changes, like diet and exercise, even during times of stress | 1🞏 | 2🞏 | 3🞏 | 4🞏 | 5🞏 |

© 2003, University of Oregon, Judith H. Hibbard, Dr. P.H. (questions 1-13)

# **SF-12® HEALTH SURVEY**

This survey asks for your views about your health. This information will help you keep track of how you feel and how well you are able to do your usual activities. Answer every question by selecting the answer as indicated. If you are unsure about how to answer a question, please give the best answer you can.

**24.** In general, would you say your health is:

1□ Excellent

2□ Very good

3□ Good

4□ Fair

5□ Poor

**VISUAL ANALOG QUALITY OF LIFE QUESTION**

**25.** On average how would you rank your quality of life over the last week?

Please circle number.

0 1 2 3 4 5 6 7 8 9 10

Worst

Imaginable

Best

Imaginable

**RAPID FOOD SCREENER**

| Meat & Snacks | Once a month or less | 2-3 times/month | 1-2 time/week | | 3-4 times/week | | 5 or more times/week | |  |
| --- | --- | --- | --- | --- | --- | --- | --- | --- | --- |
| Hamburgers, ground beef, meat burritos, tacos |  |  |  | |  | |  | |  |
| Beef or pork, such as steaks, roasts, ribs, or in sandwiches |  |  |  | |  | |  | |  |
| Fried chicken |  |  |  | |  | |  | |  |
| Hot dogs, or Polish or Italian sausages |  |  |  | |  | |  | |  |
| Cold cuts, lunch meats, ham (not low-fat) |  |  |  | |  | |  | |  |
| Bacon or breakfast sausage |  |  |  | |  | |  | |  |
| Salad dressing (not low-fat) |  |  |  | |  | |  | |  |
| Margarine, butter, or mayo on bread or potatoes |  |  |  | |  | |  | |  |
| Margarine, butter, or oil in cooking |  |  |  | |  | |  | |  |
| Eggs (not Egg Beaters or just egg whites) |  |  |  | |  | |  | |  |
| Pizza |  |  |  | |  | |  | |  |
| Cheese, cheese spread (not low-fat) |  |  |  | |  | |  | |  |
| Whole milk |  |  |  | |  | |  | |  |
| French fries, fried potatoes |  |  |  | |  | |  | |  |
| Corn chips, potato chips, popcorn, crackers |  |  |  | |  | |  | |  |
| Donuts, pastries, cake, cookies (not low-fat) |  |  |  | |  | |  | |  |
| Ice cream (not sherbet or non-fat) |  |  |  | |  | |  | |  |
| Fruits, Vegetables, and Grains | Less than once a week | Once a week | 2-3 times/week | 4-6 times/ week | | Once a day | | 2+ times/day | |
| Fruit juice, like orange, apple, grape, fresh, frozen or canned (not sodas or other drinks) |  |  |  |  | |  | |  | |
| How often do you eat any fruit, fresh or canned (not counting juice) |  |  |  |  | |  | |  | |
| Vegetable juice, like tomato juice, V-8, carrot |  |  |  |  | |  | |  | |
| Green salad |  |  |  |  | |  | |  | |
| Potatoes, any kind, including baked, mashed, or French fried |  |  |  |  | |  | |  | |
| Vegetable soup, or stew with vegetables |  |  |  |  | |  | |  | |
| Any other vegetables, including string beans, peas, corn, broccoli, or any other kind |  |  |  |  | |  | |  | |
| Fiber cereals like Raisin Bran, Shredded Wheat, or Fruit-n-Fiber |  |  |  |  | |  | |  | |
| Beans such as baked beans, pinto, kidney, or lentils (not green beans) |  |  |  |  | |  | |  | |
| Dark bread such as whole wheat or rye |  |  |  |  | |  | |  | |

**STANFORD BRIEF ACTIVITY SURVEY**

**26.** Please check the box next to the one statement that best describes the kinds of physical activity you usually performed while on the job this last year. If you are not gainfully employed outside the home but perform work around the home regularly, indicate that activity in this section.

1□ If you have no job or regular work, check here and go on to the next question.

2□ I spent most of the day sitting or standing. When I was at work, I did such things as writing, typing, talking on the telephone, assembling small parts, or operating a machine that takes very little exertion or strength. If I drove a car or truck while at work, I did not lift or carry anything for more that a few minutes each day.

3□ I spent most of the day walking or using my hands and arms in work that requires moderate exertion. When I was at work I did such things as delivering mail, patrolling on guard duty, mechanical work on automobiles or other large machines, house painting or operating a machine that requires some moderate activity work of me. If I drove a truck or lift, my job required me to lift and carry things frequently.

4□ I spent most of the day lifting or carrying heavy objects or moving most of my body in some other way. When I was at work, I did such things as stacking cargo or inventory, handling parts or materials, or I did work like that of a carpenter who builds structures or a gardener who does most of the work without machines.

5□ I spent most of the day doing hard physical labor. When I was at work I did such things as digging or chopping with heavy tools, or carrying heavy loads (bricks, for example) to the place where they are to be used. If I drove a truck or operated equipment, my job also required me to do hard physical work most of the day with only short breaks.

**27.** Please check the box next to the one statement that best describes the way you spent your leisure time during most of the last year.

1□ Most of my leisure time was spent without very much physical activity. I mostly did things like watching television, reading, or playing cards. If I did anything else, it was likely to be light chores around the house or yard or some easy-going game like bowling or catch. Only occasionally, no more than once or twice a month, did I do anything more vigorous, like jogging, playing tennis, or active gardening.

2□ Weekdays, when I got home from work, I did few active things, but most weekends I was able to get outdoors for some light exercise -- going for walks, playing a round of golf (without motorized carts), or doing some active chores around the house.

3□ Three times per week, on average, I engaged in some moderate activity, such as brisk walking or slow jogging, swimming, or riding a bike for 15 - 20 minutes or more, or I spent 45 minutes to an hour or more doing moderately difficult chores, such as raking or washing windows, mowing the lawn or vacuuming, or playing games such a doubles tennis or basketball.

4□ During my leisure time over the past year, I engaged in a regular program of physical fitness involving some kind of heavy physical activity at least three times per week. Examples of heavy physical activity are jogging, running, or riding fast on a bicycle for 30 minutes or more; heavy gardening or other chores for an hour or more; active games or sports such as handball or tennis for an hour or more; or a regular program involving calisthenics and jogging or the equivalent for 30 minute or more.

5□ Over the past year, I engaged in a regular program of physical fitness, involving some kind of heavy physical activity at least three times per week. Examples of heavy physical activity are jogging, running, or riding fast on a bicycle for 30 minutes or more; heavy gardening or other chores for an hour or more; active games or sports such as handball or tennis for an hour or more; or a regular program involving calisthenics and jogging or the equivalent for 30 minute or more, but I did it almost daily -- five or more times per week.

**TOBACCO AND ALCOHOL USE**

**28.** Do you currently smoke cigarettes or use tobacco products?

1□ Yes

2□ No

**29.** Do you currently drink alcohol at the recommended daily amount (≤ 2 glasses of wine or beer, or 1 shot of liquor)?

1□ Yes I drink the recommended amount

2□ Yes, I drink more than the recommended amount

3□ No, I do not currently drink alcohol, although I used to drink more than the recommended amount

4□ No, I do not currently drink alcohol, although I used to drink the recommended amount

5□ No I never drank alcohol

**CANCER SCREENING**

**30.** In the past year, have you had any of the cancer screening tests below? Please check all that apply.

1□ Mammography

2□ Colonoscopy

3□ Stool blood test - often called fecal occult blood test or FOBT

4□ Prostate blood test – often called PSA

5□ Digital rectal exam

**31.** Do you plan in the next one year to have any of the cancer screening tests below? Please check all that apply.

1□ Mammography

2□ Colonoscopy

3□ Stool blood test - often called fecal occult blood test or FOBT

4□ Prostate blood test – often called PSA

5□ Digital rectal exam

INFORMATION SEEKING

32. The most recent time you looked for information about health or medical topics, where did you go first?

[Mark only one.]

□ Books

□ Brochures, pamphlets, etc.

□ Cancer organization

□ Family

□ Friend/co-worker

□ Doctor or healthcare provider

□ Internet

□ Library

□ Magazines

□ Newspapers

□ Telephone information number

□ Complementary, alternative, or unconventional practitioner

□ Other (fill in the blank)____________________________________________________

33. Overall, how confident are you that you could get health-related advice or information if you needed it?

□ Completely confident

□ Very confident

□ Somewhat confident

□ A little confident

□ Not confident at all
