# Supplementary material for: Implementation-effectiveness trial of systematic family health history based risk assessment and impact on clinical disease prevention and surveillance activities
Source: BMC Health Serv Res. 2022 Dec 6;22:1486. doi: 10.1186/s12913-022-08879-2 (PMC9727967; doi:10.1186/s12913-022-08879-2)
Supplement: Supplementary file 4 — Additional file 4. [file 12913_2022_8879_MOESM4_ESM.docx]

Date: _____________

Age: _____________ (if 90 or older, use 90+)

Clinic: ______________

Gender: 1€ Male

2€ Female

Race: 1€ White 4€ American Indian/Alaskan Native

2€ Black or African American 5€ Pacific Islander/Native Hawaiian

3€ Asian 6€ Other (specify) __________

Ethnicity: 1€ Hispanic or Latino

2€ Not Hispanic or Latino

Years in Practice: ______

### What is the practice setting in which you spend the majority of your time?

### Academic Center

### Non-Academic Hospital

### Private Practice (group or solo)

### Community Health Center

### Nursing home or long term care facility

### ER or acute care center in academic setting

### ER or acute care center not in academic setting

- Other____________________

Profession:

- Physician
- Physician’s Assistant
- Nurse/Advanced Nurse Practitioner
- Genetic counselor
- Pharmacist
- Other (specify)________________

What is your primary practice specialty? (if you have more than one, please choose the one where you spend the most time)

- Allergy/Immunology
- Cardiology
- Dermatology
- Endocrinology/Diabetes/Metabolism
- Family Medicine
- Gastroenterology
- Geriatrics
- Internal Medicine
- Hematology/Oncology
- Infectious Diseases
- Medical Genetics
- Neurology
- Obstetrics/Gynecology
- Ophthalmology
- Pediatrics
- Pulmonary
- Psychiatry
- Rheumatology
- Surgery
- Other (specify)______________________________

1. Is using computerized family history an acceptable substitute for a physician collecting it or asking family history on an intake form (check one)?

1€ Yes

2€ No

2. Would you recommend having risk reports (family history and risk score) for all patients?

1€ Yes

2€ No

3. Would you recommend that your peers be able to use pre-appointment computerized risk collection in their clinics?

1€ Yes

2€ No

4. Did your patients report any anxiety about undergoing risk scoring (such as fear of discrimination or fears about their own health)?

1€ Yes

2€ No

5. Did you experience any anxiety related to the calculation of the risk scores or the use of computerized system for collecting and generating the scores?

1€ Yes

2€ No

6. Did generating risk scores negatively impact the clinic workflow or patient flow in your clinic?

1€ Yes

2€ No

7. On average how much time do you spend discussing risk score results with patients?

Drop box with 1-30 as options

8. Have the risk scores or genetic test results altered how you practice (for example have you changed your plan of care for any of your patients due to report information) for study participants?

1€ Yes

2€ No

a. If yes, how did it change (check all the boxes that apply)?

1€ Changed cancer screening intervals

2€ Changed cancer screening method

3€ Changed medications patient was taking

4€ Changed recommendations for preventative behaviors (such as avoiding prolonged flights)

5€ Referred to a genetic counselor

6€ Recommended family members be screened

7€ Changed dietary recommendations

8€ Changed exercise recommendations

9€ More strongly encouraged smoking cessation

9. Have the risk reports made your practice (check one):

1€ Easier

2€ No change

3€ More complicated

10. To what extent to you agree or disagree with the following statements:

- 1. I think collecting family history is more important now compared to when the project started.

1€ Strongly agree

2€ Somewhat Agree

3€ Agree

4€ Disagree

5€ Strongly Disagree

- 1. I think calculating risk scores is more important now compared to when the project started.

1€ Strongly agree

2€ Somewhat Agree

3€ Agree

4€ Disagree

5€ Strongly Disagree

- 1. I understand risk scores better now than when the project started.

1€ Strongly agree

2€ Somewhat Agree

3€ Agree

4€ Disagree

5€ Strongly Disagree

11. In looking at how to incorporate this type of risk collection into routine practice,

1. Would it be helpful to have someone with genetic experience (not necessarily a cancer genetic specialist) available to review family histories (circle one)?

1€ Yes

2€ No

3€ Not sure

1. Would it be to have a relationship with a genetic specialist (circle one)

1€ Yes

2€ No

3€ Not Sure

1. How often had you referred to a genetic specialist **prior** to this study?

1€ Never

2€ 1-5 times

3€ 6-15 times

4€ > 15 times

12. Were there times when you would have liked to follow a MeTree recommendation but were unable due to due to limited resources or other process-related difficulties? If so, please select each recommendation you had a problem with and **describe the problem**.

1€ Breast MRI

2€ Breast Cancer chemoprevention

3€ GYN referral for discussion of ovarian cancer screening

3€ Early or more frequent colon cancer screening

3€ low dose CT for lung cancer screening

3€ Genetic counseling

3€ Genetic Testing

3€ Aortic ultrasound

3€ Calcium Scoring CT

3€ Carotid Intimal Medical Thickening ultrasound

3€ high resolution CRP

Please describe the problem you had with the selected recommendations chosen

|  |
| --- |

13a. How frequently did you disagree with a risk report recommendation?

1€ Always

2€ Most of the time

3€ Half the time

4€ Rarely

5€ Never

13b. If you disagreed with a risk report recommendation select which recommendations you disagree with and describe **why**.

1€ Breast MRI screening

2€ Breast cancer chemoprevention

3€ Colon cancer screening frequency or age of onset

4€ Lung cancer screening

5€ Genetic counseling referral

6€ Genetic testing

7€ Cardiovascular risk score results and recommendations

8€ AAA screening

9€ Aspirin for stroke prevention

10€ Aspirin for myocardial infarction prevention

Please describe why you disagree with the selected recommendations

|  |
| --- |

14. In regards to the format in which the risk scores are reported:

1. Do you feel patient communication was enhanced by the reports?

1€ Yes

2€ No

1. Do you feel your patients were more motivated to change their behavior after receiving risk scores?

1€ Yes

2€ No

1. Do you feel your patients were more motivated to change their behavior after receiving genetic tests?

1€ Yes

2€ No

3€ No patient received genetic tests
